# Theoretical illustration of the effect of 1-ethyl-pyridinium trifluoroacetate ionic liquid in the enhancement of the Diels–Alder reaction of isoprene with acrylic acid

**DOI:** 10.1186/1758-2946-6-S1-P58

**Published:** 2014-03-11

**Authors:** Hafida Chemouri, Sidi Mohamed Mekelleche

**Affiliations:** 1 Laboratory of Applied Thermodynamics and Molecular Modeling, Department of Chemistry, Faculty of Science, University A. Belkaïd, B. P. 119, Tlemcen, 13000, Algeria; 2 Preparing School in Sciences and Techniques of Tlemcen, BP 165 RP Bel Horizon, 13000, Tlemcen, Algeria

## 

**Figure 1:**
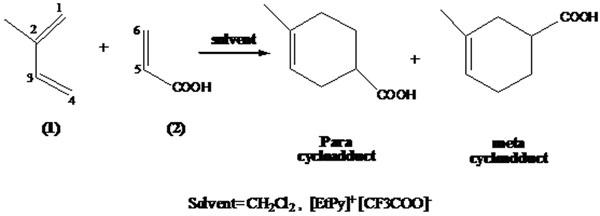
Diels–Alder reactions of isoprene (1) and acrylic acid (2).

The Diels–Alder reaction is a powerful tool in organic synthesis and in the chemical industry. Recently an increased attention has been focused on the development of green methods for the purpose of improving rate and selectivity of this reaction. In recent years, ionic liquids (ILs) have gained a lot of attention as green solvents in organic synthesis and other chemical processes. This is mainly due to their favorable inherent properties such as chemical and thermal stability, no measurable vapor pressure, nontoxicity, nonflammability, catalytic ability, high polarity, ease to recycle, etc. Diels–Alder reactions of isoprene (1) and acrylic acid (2) (Figure 1) have been investigated in pyridinium based ILs. The ionic liquid 1-ethyl-pyridinium trifluoroacetate [EtPy]^+^ [CF3COO]^-^ is found to be an excellent reaction solvent with significantly increased rate for this reaction compared to organic solvent.

In order to confirm this experimental finding, theoretical investigation of the regioselectivity meta/para of this Diels Alder reaction have been carried out. The calculations have been performed in gas phase, in CH2Cl2 organic solvent and in 1-ethyl-pyridinium trifluoroacetate protic IL. Asynchronous concerted mechanisms yielding to the formation of the para regioisomers as the major cycloadducts is shown by the analysis of the relevant stationary points of the potential energy surface and intrinsic reaction coordinate calculations carried out in gas phase. The calculation of activation and reaction energies indicates that the para cycloadducts are favored both kinetically and thermodynamically. The calculations, performed using explicit solvent model, show a considerable decrease of the activation barrier in the IL in comparison with gas phase and CH2Cl2. The obtained results put in evidence the importance of hydrogen bonding formed between the IL and the dienophile fragment in the promotion of this Diels-Alder reaction. The calculations are carried out with the Gaussian 09 suite of programmes using the B3PW91 exchange-correlation functionals together with the 6–31G(d,p) basis set and the obtained results are in good agreement with experimental outcomes.
